# Comparative Antimicrobial Resistance Patterns in Extraintestinal *Escherichia coli* Isolates from Humans and Broiler Chickens in the Meknes Region, Morocco: A One Health Study

**DOI:** 10.3390/antibiotics15101039

**Published:** 2026-10-21

**Authors:** Hakim Ennaciri, Amin Regragui, Driss Bengoumi, Naoufal Rahmatallah, Rabii El Bahraouy, Mohammed Sbiti, Mohamed Reda El Rhaffouli, Ikhlass El Berbri, Hicham El Rhaffouli, Ouafaa Fassi Fihri

**Affiliations:** 1 Department of Veterinary Pathology and Public Health, Hassan II Agronomic and Veterinary Institute, B.P. 6202, Madinat Al Irfane, Rabat 10101, Morocco; regraguiamin90@gmail.com (A.R.); n.rahmatallah@gmail.com (N.R.); i.elberbri@iav.ac.ma (I.E.B.); o.fassifihri@iav.ac.ma (O.F.F.); 2 Faculty of Sciences, Moulay Ismail University, B.P. 11201, Zitoune, Meknes 50050, Morocco; bengoumi@yahoo.fr; 3 Faculty of Medicine and Pharmacy, Sidi Mohamed Ben Abdellah University, Fez 30003, Morocco; rabii-far@hotmail.com (R.E.B.); sbiti.simo@yahoo.fr (M.S.); 4 Bacteriology Department, Moulay Ismail Military Hospital, Meknes 50000, Morocco; 5 National Higher School of Chemistry, IBN Tofail University, Kenitra 14000, Morocco; mohamed.reda.elrhaffouli@uit.ac.ma; 6 Veterinary Division, Military Health Service, Rabat 10045, Morocco; elrhaffoulihicham@live.fr

**Keywords:** antimicrobial resistance, *Escherichia coli*, broiler chickens, One Health, β-lactam resistance, ESBL

## Abstract

**Background/Objectives:** Antimicrobial resistance (AMR) is a major One Health challenge affecting both human and animal health. Comparative studies investigating resistance patterns across reservoirs remain limited in Morocco. This study compared antimicrobial resistance profiles and presumptive β-lactam resistance phenotypes of extraintestinal *Escherichia coli* isolates from humans and broiler chickens. **Methods:** A comparative cross-sectional study was conducted using 874 non-duplicate human-derived *E. coli* isolates and 142 broiler-derived *E. coli* isolates. Antimicrobial susceptibility testing was performed using the disk diffusion method according to CA-SFM/EUCAST 2024 guidelines. Resistance rates and presumptive β-lactam resistance phenotypes were compared between reservoirs. **Results:** Broiler isolates exhibited significantly higher resistance to amoxicillin (93.0% vs. 65.5%), ticarcillin (92.9% vs. 64.1%), amoxicillin–clavulanic acid (75.2% vs. 48.1%), fluoroquinolones, trimethoprim–sulfamethoxazole, fosfomycin, and gentamicin (all *p* < 0.05). In contrast, resistance to cephalosporins was significantly higher among human isolates, whereas resistance to cefoxitin and carbapenems was detected exclusively among human isolates. The presumptive IRT-like phenotype was markedly more prevalent in broiler isolates (66.9% vs. 26.9%; OR = 5.50; *p* < 0.001), while the presumptive AmpC and carbapenem-resistant phenotypes were identified only in human isolates. Conversely, the wild-type phenotype was significantly more frequent among human isolates (33.1% vs. 5.6%; OR = 0.12; *p* < 0.001). The prevalence of the presumptive ESBL phenotype was comparable between reservoirs (12.1% vs. 8.5%; *p* = 0.258). **Conclusions:** Human-derived and broiler-derived *E. coli* isolates exhibited distinct antimicrobial resistance patterns and β-lactam resistance profiles. Resistance levels were significantly higher among broiler isolates for most antimicrobial agents tested, whereas resistance to cephalosporins was significantly higher among human isolates, with cefoxitin and carbapenem resistance detected exclusively in the latter. Human isolates displayed a greater diversity of presumptive β-lactam resistance phenotypes, whereas broiler isolates were largely dominated by the presumptive IRT-like phenotype. The detection of isolates with a presumptive ESBL phenotype among broilers despite the absence of documented routine use of extended-spectrum cephalosporins highlights the potential contribution of horizontal gene transfer and co-selection. Further molecular and antimicrobial consumption studies are warranted to clarify the drivers of resistance dissemination within a One Health framework.

## 1. Introduction

Antimicrobial resistance (AMR) has emerged as a major global threat to both human and animal health in the 21st century [1]. Recent estimates indicate that by 2050, approximately 1.91 million deaths per year could be directly attributable to antimicrobial resistance, with more than 8 million deaths associated with resistant infections worldwide [2]. The burden of AMR is particularly pronounced in low- and middle-income countries, where high antimicrobial consumption and limited surveillance systems may contribute to the emergence and spread of resistant bacteria [3].

In Morocco, hospital-based studies have reported substantial mortality associated with infections caused by multidrug-resistant (MDR) bacteria. An attributable mortality rate of approximately 12% has been documented among patients admitted to intensive care units with MDR infections, highlighting the significant clinical burden of AMR at the national level [4].

Beyond the clinical setting, antimicrobial resistance is increasingly reported in food-producing animals. In Morocco, poultry constitutes a major source of animal protein, with a per capita consumption of approximately 18.78 kg per year, representing nearly 57% of total meat intake, according to FAOSTAT Food Balance Sheets Data [5]. This sector is characterized by intensive production systems in which antimicrobials are commonly used [6]. These characteristics support the selection of broiler chickens as a relevant animal reservoir for comparison with human-derived isolates. Several studies have reported high resistance rates to multiple antibiotic classes among *Escherichia coli* isolates from broilers worldwide, with similar trends observed in Morocco [7,8], raising concerns about the potential role of poultry as a reservoir of antimicrobial resistance.

Within the framework of the One Health approach, which recognizes the interconnectedness of human, animal, and environmental health, comparative analyses of antimicrobial resistance patterns across different reservoirs are essential. However, the extent to which resistance profiles in human and poultry *E. coli* isolates reflect shared selective pressures or independent evolutionary dynamics remains insufficiently understood. Therefore, the present study aimed to compare antimicrobial resistance profiles of *E. coli* isolates from human and broiler sources and to explore similarities and differences in resistance patterns within a One Health framework, without inferring direct transmission pathways.

Despite increasing reports of antimicrobial resistance in both clinical and veterinary settings in Morocco, direct comparative studies focusing on extraintestinal *E. coli* isolates from humans and poultry remain limited. Investigating whether resistance patterns converge or diverge between these reservoirs is essential to inform integrated antimicrobial stewardship strategies.

## 2. Results

During the study period, antimicrobial susceptibility data from 874 human *Escherichia coli* isolates were obtained from routine diagnostic testing performed at the bacteriology laboratory of the Moulay Ismail Military Hospital, Meknes, Morocco.

In parallel, antimicrobial susceptibility testing was performed on 142 *E. coli* isolates recovered from broiler chickens originating from commercial farms in the Fez–Meknes region. The antimicrobial panel included a broad range of antibiotic classes, including agents primarily used in human medicine.

**Figure 1:**
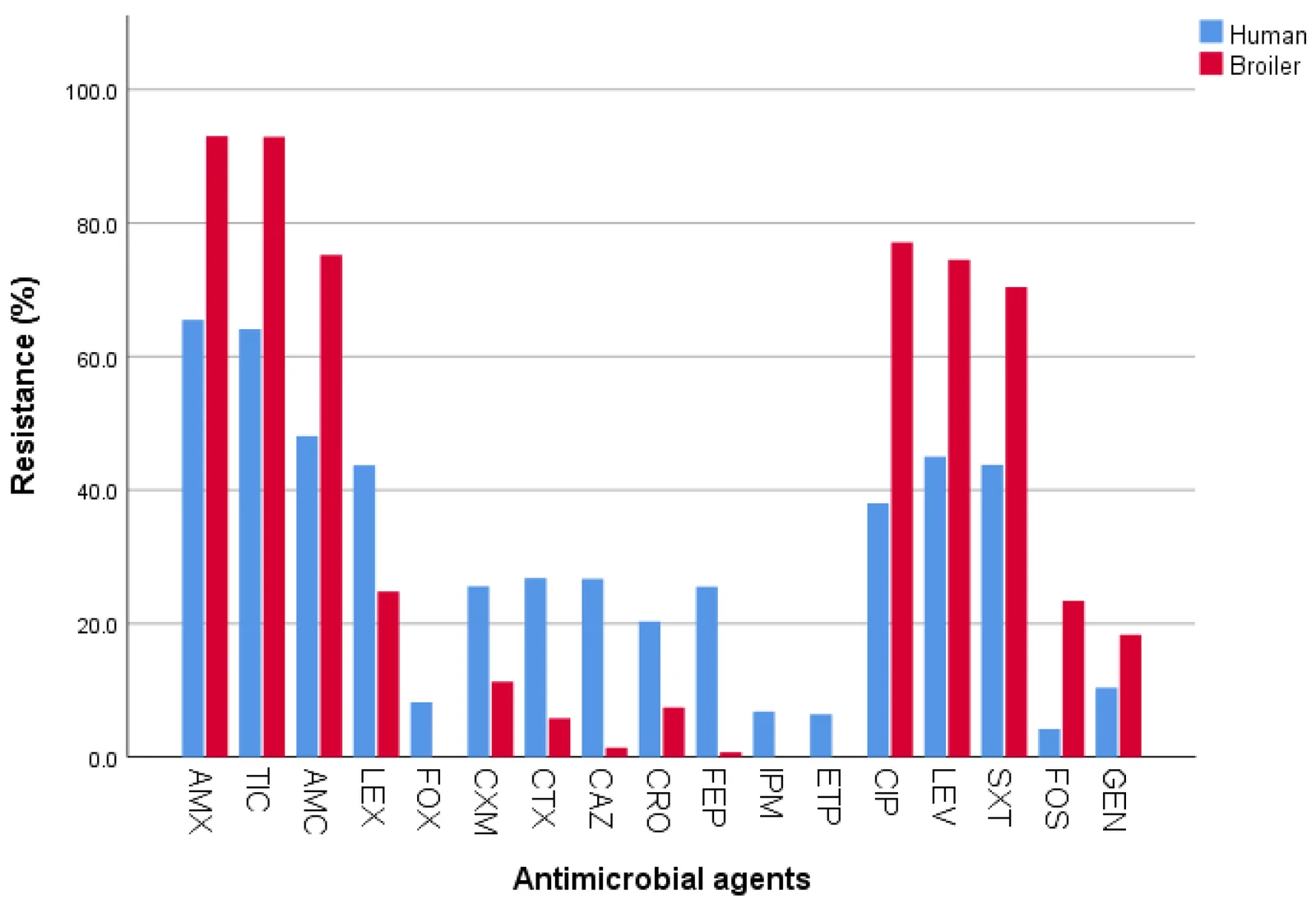
Comparison of antimicrobial resistance rates (%) between human and broiler *Escherichia coli* isolates.

The comparative distribution of antimicrobial resistance rates between human and broiler isolates is presented in Table 1 and illustrated in Figure 1, together with the corresponding odds ratios (ORs) and *p*-values.

Among the tested antimicrobial agents, resistance to aminopenicillins and penicillin combinations was significantly higher in broiler isolates than in human isolates, particularly for amoxicillin (93.0% vs. 65.5%; OR = 6.96; *p* < 0.001) and ticarcillin (92.9% vs. 64.1%; OR = 7.32; *p* < 0.001). Similarly, resistance to amoxicillin–clavulanic acid was significantly higher in broilers (75.2% vs. 48.1%; OR = 3.27; *p* < 0.001).

In contrast, resistance to cephalosporins, including cefotaxime, ceftazidime, ceftriaxone, and cefepime, was significantly higher in human-derived isolates (Table 1).

Notably, resistance to cefoxitin (8.2% vs. 0.0%; *p* < 0.001), imipenem (6.8% vs. 0.0%; *p* = 0.001), and ertapenem (6.4% vs. 0.0%; *p* = 0.002) was observed exclusively in human-derived isolates, whereas no resistance to these antimicrobial agents was detected among broiler isolates.

Resistance to fluoroquinolones was markedly higher in broiler isolates, with resistance to ciprofloxacin reaching 77.1% compared to 38.0% in human-derived isolates (OR = 5.51; 95% CI: 3.63–8.37; *p* < 0.001), while resistance to levofloxacin was 74.5% versus 45.0%, respectively (OR = 3.57; 95% CI: 2.38–5.37; *p* < 0.001).

Broiler isolates also exhibited significantly higher resistance rates to trimethoprim–sulfamethoxazole (70.4% vs. 43.8%; OR = 3.05; 95% CI: 2.07–4.50; *p* < 0.001) and fosfomycin (23.4% vs. 4.2%; OR = 6.93; 95% CI: 3.68–13.08; *p* < 0.001).

A moderate but significant increase in gentamicin resistance was observed in broilers compared to human-derived isolates (18.3% vs. 10.4%; OR = 1.93; 95% CI: 1.19–3.11; *p* = 0.006).

Given the major role of β-lactam antibiotics in both human and veterinary medicine, the distribution of presumptive β-lactam resistance phenotypes was analyzed in *E. coli* isolates from human and broiler sources (Table 2).

The distribution of presumptive β-lactam resistance phenotypes differed markedly between the two reservoirs. The IRT-like phenotype was significantly more prevalent among broiler isolates than among human isolates (66.9% vs. 26.9%; OR = 5.50, 95% CI: 3.76–8.04; *p* < 0.001). In contrast, the wild-type phenotype was significantly more frequent in human isolates (33.1% vs. 5.6%; OR = 0.12, 95% CI: 0.06–0.25; *p* < 0.001).

The prevalence of the penicillinase phenotype was identical in both reservoirs (17.6% in each group; OR = 1.00, 95% CI: 0.63–1.58; *p* = 1.000). No significant difference was observed for the ESBL phenotype, which was detected in 12.1% of human isolates and 8.5% of broiler isolates (OR = 0.67, 95% CI: 0.36–1.25; *p* = 0.258). AmpC and carbapenem-resistant phenotypes were identified exclusively in human isolates, accounting for 2.2% and 6.1% of isolates, respectively, whereas no such phenotypes were detected in broiler isolates (Table 2).

## 3. Discussion

The present study provides a comparative analysis of antimicrobial resistance patterns in extraintestinal *Escherichia coli* isolates from human and broiler sources in Morocco, revealing marked differences in resistance profiles and underlying β-lactam resistance phenotypes within a One Health framework.

A major finding of this study is the marked divergence in β-lactam resistance between human- and broiler-derived isolates. Regarding aminopenicillins and carboxypenicillins, broiler-derived isolates exhibited significantly higher resistance to amoxicillin (93.0% vs. 65.5%; OR = 6.96; *p* < 0.001) and ticarcillin (92.9% vs. 64.1%; OR = 7.32; *p* < 0.001). The high amoxicillin resistance observed among broiler-derived *E. coli* was consistent with previous Moroccan studies, which reported rates of 90.9% and 94.62% in poultry isolates [8,10]. Similarly, the resistance rate observed among human-derived isolates (65.5%) closely agrees with the 65% previously reported by El Bouamri et al. among urinary *E. coli* isolates in Morocco [11]. More recent Moroccan surveillance data have confirmed that resistance to aminopenicillins remains high among urinary *E. coli* isolates [12]. Comparably high levels of penicillin resistance have also been reported in neighboring Algeria, where *E. coli* isolates recovered from chickens with colibacillosis showed resistance rates of 83.3% to amoxicillin and 80.5% to ticarcillin [13].

Notably, despite the marked differences in resistance to individual penicillins between the two populations, the presumptive penicillinase phenotype was observed at the same frequency in human- and broiler-derived isolates (17.6% in both populations; OR = 1.00; 95% CI: 0.63–1.58; *p* = 1.000). The similar frequency of this phenotype in the two populations, together with previous molecular evidence of *bla*_TEM-1_ in human- and poultry-derived *E. coli* in Morocco [14,15], provides support for the hypothesis that narrow-spectrum β-lactam resistance determinants may be broadly distributed across *E. coli* populations from different reservoirs rather than being restricted to a particular host population. This hypothesis is also consistent with findings from other geographical settings. Borges et al. identified *bla*_TEM-1_ as the most prevalent β-lactamase gene among both human- and chicken-derived *E. coli*, occurring in 92% and 67%, respectively, of isolates carrying β-lactamase genes [16]. More recently, *bla*_TEM-1_ predominated among β-lactam-resistant poultry *E. coli* in South Korea, where it was detected in 81.8% of isolates [17]. However, in the absence of molecular characterization, the observed phenotype cannot be attributed to specific β-lactamases, and the potential contribution of other resistance mechanisms, such as reduced outer-membrane permeability or increased efflux, cannot be excluded.

A similar reservoir-specific difference was observed for amoxicillin–clavulanic acid, with resistance being significantly higher among broiler-derived than human-derived isolates (75.2% vs. 48.1%; OR = 3.27; *p* < 0.001). This finding is particularly noteworthy in the Moroccan regulatory context, where amoxicillin-containing veterinary medicinal products are authorized for use in poultry, whereas no amoxicillin–clavulanic acid-containing product is listed for this species in the national positive list of authorized veterinary medicinal products [18]. Furthermore, Moroccan regulations restrict the administration of veterinary medicinal products to the animal species specified in their marketing authorization [19].

This marked difference in amoxicillin–clavulanic acid resistance was paralleled by a significantly higher frequency of the presumptive inhibitor-resistant β-lactamase phenotype (IRT-like) among broiler-derived than human-derived isolates (66.9% vs. 26.9%; OR = 5.50; 95% CI: 3.76–8.04; *p* < 0.001). This phenotypic pattern can be interpreted within the framework described by Bonnet, who distinguished a high-level penicillinase phenotype, in which strong penicillinase activity may reduce susceptibility to β-lactam/β-lactamase inhibitor combinations, from an inhibitor-resistant penicillinase phenotype characterized by resistance to aminopenicillins, amoxicillin–clavulanate, and carboxypenicillins, with preserved susceptibility to extended-spectrum cephalosporins and carbapenems [20]. Consistent with this framework, previous studies have demonstrated that hyperproduction of narrow-spectrum β-lactamases, particularly TEM-1, can reduce susceptibility to amoxicillin–clavulanic acid by limiting the inhibitory effect of clavulanate [21,22]. Penicillinase hyperproduction may therefore represent one possible explanation for the high frequency of presumptive IRT-like profiles observed among broiler-derived isolates. However, this interpretation remains hypothetical because phenotypic testing cannot distinguish penicillinase hyperproduction from inhibitor-resistant β-lactamases or other potential resistance mechanisms. Consequently, the molecular basis of these IRT-like profiles remains to be established.

In contrast to the pattern observed for penicillins and amoxicillin–clavulanic acid, resistance to extended-spectrum cephalosporins was significantly more frequent among human-derived isolates across all tested agents, including cefotaxime (26.8% vs. 5.8%), ceftazidime (26.7% vs. 1.4%), ceftriaxone (20.3% vs. 7.4%), and cefepime (25.5% vs. 0.7%) (all *p* < 0.001). Among human-derived isolates, these findings are broadly consistent with national Moroccan surveillance data from 2022, which reported resistance rates of 19% to cefotaxime, ceftriaxone, and ceftazidime and 20% to cefepime among *E. coli* isolates [23]. Directly comparable Moroccan data on resistance to extended-spectrum cephalosporins among broiler-derived *E. coli* remain limited, as available studies differ in sampling origin, study design, and antimicrobial panels.

Despite these marked differences in resistance to individual extended-spectrum cephalosporins, the presumptive ESBL phenotype was detected in both populations, with a slightly higher frequency among human-derived than broiler-derived isolates (12.1% vs. 8.5%); however, this difference was not statistically significant (OR = 0.67; 95% CI: 0.36–1.25; *p* = 0.258). The higher resistance to extended-spectrum cephalosporins among human-derived isolates, despite the absence of a significant difference in the presumptive ESBL phenotype, may partly reflect the broader diversity of β-lactam resistance phenotypes observed in the human population, including presumptive AmpC and carbapenem-resistant phenotypes, which were detected exclusively among human-derived isolates. Importantly, the absence of a significant difference in the frequency of the presumptive ESBL phenotype between the two reservoirs should be interpreted in the context of markedly different overall β-lactam phenotype distributions. Wild-type isolates were substantially more frequent among human-derived than broiler-derived isolates (33.1% vs. 5.6%), whereas the presumptive IRT-like phenotype predominated among broiler-derived isolates (66.9% vs. 26.9%).

In addition, as previously noted for ticarcillin and amoxicillin–clavulanic acid, the detection of presumptive ESBL-producing *E. coli* among broiler-derived isolates, despite the absence of authorized use of third- and fourth-generation cephalosporins in Moroccan poultry production [18,19], further highlights the complexity of antimicrobial resistance dynamics. Previous studies have shown that ESBL-encoding genes are frequently carried on plasmids and may disseminate through horizontal gene transfer, while their association with other antimicrobial resistance determinants may facilitate co-selection and contribute to their persistence even in the absence of direct selective pressure from these antimicrobial classes [24,25]. These mechanisms could potentially contribute to the occurrence and persistence of presumptive ESBL phenotypes among broiler-derived isolates in the present study; however, this hypothesis cannot be confirmed in the absence of molecular characterization.

This reservoir-specific pattern was also observed for cefoxitin and carbapenems. Resistance to cefoxitin, imipenem, and ertapenem was detected exclusively among human-derived isolates, at rates of 8.2%, 6.8%, and 6.4%, respectively, whereas no resistance to these agents was detected among broiler-derived isolates. Consistent with these susceptibility patterns, the presumptive AmpC and carbapenem-resistant phenotypes were identified only among human-derived isolates, at frequencies of 2.2% and 6.1%, respectively, whereas neither phenotype was detected among broiler-derived isolates.

The absence of these phenotypes among broiler-derived isolates is noteworthy in the Moroccan regulatory context, where third- and fourth-generation cephalosporins are not listed among veterinary medicinal products authorized for poultry production, and carbapenems are prohibited for use in food-producing animals [18,19]. From a methodological perspective, cefoxitin was included in the antimicrobial susceptibility testing panel as a phenotypic marker for AmpC detection rather than for therapeutic purposes. AmpC β-lactamases confer resistance to cephamycins and may arise from chromosomal derepression or plasmid-mediated acquisition [26], whereas carbapenem resistance may involve carbapenemase production and/or reduced membrane permeability associated with porin loss, often in combination with β-lactamase activity [27].

The exclusive detection of presumptive AmpC and carbapenem-resistant phenotypes among human-derived isolates may reflect differences in antimicrobial exposure and selective pressures between the two reservoirs. Previous exposure to third-generation cephalosporins has been associated with an increased risk of infection with AmpC-producing *E. coli* or *Klebsiella pneumoniae*, while carbapenem exposure has been identified as an important risk factor for the acquisition of carbapenem-resistant Enterobacterales [28,29]. These associations provide a plausible epidemiological context for the phenotypic differences observed in the present study; however, individual antimicrobial exposure was not assessed, and the underlying resistance mechanisms cannot be determined in the absence of molecular characterization.

Taken together, these findings reveal markedly different distributions of presumptive β-lactam resistance phenotypes between the two reservoirs. Broiler-derived isolates were characterized by a predominance of the presumptive IRT-like phenotype (66.9%) and a low proportion of wild-type isolates (5.6%), whereas human-derived isolates displayed a more diverse phenotypic distribution, with a substantially higher proportion of wild-type isolates (33.1%) and the exclusive detection of presumptive AmpC and carbapenem-resistant phenotypes. These contrasting phenotypic profiles may reflect differences in antimicrobial exposure and selective pressures between the two reservoirs. However, in the absence of molecular characterization, the observed phenotypes cannot be attributed to specific resistance mechanisms. The small proportion of indeterminate profiles in both populations (2.1% in human-derived and 1.4% in broiler-derived isolates) reflects susceptibility patterns that could not be assigned to any of the predefined phenotypic categories and should not be interpreted as a distinct resistance mechanism.

Broiler-derived isolates showed markedly higher resistance to fluoroquinolones, with ciprofloxacin resistance reaching 77.1% vs. 38.0% (OR = 5.51; *p* < 0.001) and levofloxacin 74.5% versus 45.0% (OR = 3.57; *p* < 0.001). Among human-derived isolates, these rates were broadly consistent with data from the Moroccan national antimicrobial resistance surveillance report, which documented ciprofloxacin and levofloxacin resistance rates of 33% and 30%, respectively, among clinical *E. coli* isolates [23]. Previous European surveillance analyses have demonstrated significant positive associations between fluoroquinolone consumption in humans and fluoroquinolone resistance in invasive *E. coli* isolates throughout 2019–2021 [30]. High fluoroquinolone resistance has also been reported among poultry-derived *E. coli* in Morocco. Rahmatallah et al. reported 75.9% resistance to enrofloxacin among avian *E. coli* isolates, a level close to the ciprofloxacin and levofloxacin resistance rates observed in our broiler-derived isolates [10], while Oubouyahia et al. reported resistance rates of 82.8% to flumequine and 48.4% to enrofloxacin among *E. coli* isolates from Moroccan broilers [8]. Enrofloxacin has also been reported among the most commonly used antibacterials in Moroccan broiler flocks [6]. Taken together, these findings are compatible with the hypothesis that fluoroquinolone use may contribute to the selective pressures operating in both human and poultry settings [7,30]; however, antimicrobial exposure was not directly assessed in the present study.

In addition, resistance to trimethoprim–sulfamethoxazole was significantly higher among broiler-derived than human-derived isolates (70.4% vs. 43.8%; OR = 3.05; *p* < 0.001). This pattern may be consistent with co-selection mechanisms, as genes conferring resistance to trimethoprim and sulfamethoxazole can be genetically linked on mobile genetic elements, including integrons, transposons, and transferable plasmids [31].

Gentamicin resistance was also significantly higher among broiler-derived than human-derived isolates (18.3% vs. 10.4%; *p* = 0.006), despite the absence of documented routine use of gentamicin in Moroccan poultry production [18]. One possible explanation is indirect selection associated with exposure to other aminoglycosides, such as neomycin, which is marketed for poultry use in Morocco [18]. Aminoglycoside resistance may involve several aminoglycoside-modifying enzymes, and resistance determinants can occur on mobile genetic elements [32,33], providing support for the hypothesis that exposure to other aminoglycosides and co-selection may contribute to the persistence of gentamicin resistance. However, exposure to neomycin or other aminoglycosides and the underlying resistance mechanisms were not investigated in the present study. Furthermore, resistance determinants may persist even in the absence of direct selective pressure when associated with limited fitness costs or compensatory adaptations [34,35].

Fosfomycin resistance was significantly higher among broiler-derived than human-derived isolates (23.4% vs. 4.2%; OR = 6.93; 95% CI: 3.68–13.08; *p* < 0.001). Given that fosfomycin has been prohibited for use in food-producing animals in Morocco since late 2023 [19], the resistance observed among broiler-derived isolates may reflect, at least in part, the persistence of resistance determinants selected before the implementation of this regulatory restriction. In addition, plasmid-mediated fosfomycin resistance genes, particularly *fosA*-type genes, may occur on multidrug-resistance plasmids together with other resistance determinants, potentially facilitating their co-selection and persistence under selective pressure from other antimicrobial classes [36]. However, previous fosfomycin exposure and the molecular mechanisms underlying the observed resistance were not investigated in the present study; therefore, these explanations remain hypothetical.

Taken together, these findings highlight distinct, reservoir-specific antimicrobial resistance profiles in *E. coli*, which may reflect differences in antimicrobial exposure and selective pressures between human and poultry settings. Broiler-derived isolates exhibited particularly high resistance to several antimicrobial classes, whereas human-derived isolates showed a broader diversity of presumptive β-lactam resistance phenotypes, including the exclusive detection of presumptive AmpC and carbapenem-resistant phenotypes. These contrasting patterns underscore the importance of coordinated antimicrobial stewardship and surveillance across human and animal sectors within a One Health framework.

More broadly, historical antimicrobial exposure, co-selection, and the persistence of resistance determinants within bacterial populations may also contribute to the observed resistance patterns, highlighting the potential influence of factors beyond current antimicrobial use.

This study has several limitations. Resistance phenotypes were inferred from antimicrobial susceptibility patterns and phenotypic testing, including a clavulanate synergy test for ESBL detection, but were not confirmed by molecular methods, which may have resulted in the misclassification of certain resistance mechanisms. In addition, the lack of detailed antimicrobial-use and farm-level epidemiological data, including flock characteristics and management practices, limits the ability to directly link the observed resistance patterns to specific selective pressures. Furthermore, because broiler-derived isolates were recovered exclusively from birds submitted for necropsy with lesions compatible with colibacillosis, the resistance frequencies observed in this population should not be generalized to apparently healthy broilers or to the general broiler population. Finally, the absence of molecular typing or genomic characterization precludes assessment of genetic relatedness among isolates and therefore does not allow inference of inter-reservoir transmission. The present study also did not assess whether resistance in broiler-derived *E. coli* was associated with human infections or clinical outcomes. Moreover, the single-hospital origin of the human isolates limits the generalizability of these findings to other healthcare and community settings. Accordingly, the One Health dimension of this study should be interpreted as a comparative assessment of phenotypic resistance patterns across human and broiler reservoirs within the same regional context, rather than as evidence of genetic relatedness or transmission between these populations.

## 4. Materials and Methods

### 4.1. Study Design

This comparative cross-sectional study was conducted over a one-year period, from January to December 2025, to compare antimicrobial resistance profiles of *Escherichia coli* isolates from humans and broiler chickens within a One Health framework.

In the human dataset, stool-derived isolates represented only a small proportion (1.8%) of samples subjected to antimicrobial susceptibility testing. To avoid potential bias related to the inclusion of predominantly commensal intestinal isolates and to ensure comparability with broiler-derived isolates obtained from extraintestinal sites, stool-derived isolates were excluded.

Human-derived *E. coli* isolates included in the study were predominantly recovered from urinary and other extraintestinal clinical specimens. Broiler-derived *E. coli* isolates were recovered from extraintestinal organs, including the liver, heart, lungs, air sacs, and yolk sac, during necropsy of broilers presenting lesions compatible with colibacillosis [37].

Because molecular characterization of virulence-associated markers was not performed, the isolates were not assigned to specific pathogenic *E. coli* pathotypes such as ExPEC or APEC. Instead, the neutral terms “human-derived *E. coli*” and “broiler-derived *E. coli*” were used throughout the study to describe the origin of the isolates. This approach allowed comparison of antimicrobial resistance profiles between the two reservoirs without implying a definitive molecular pathotype classification [38,39].

### 4.2. Study Settings and Bacterial Isolates

#### 4.2.1. Human-Derived *E. coli* Isolates

A total of 874 non-duplicate *E. coli* isolates were recovered from routine diagnostic microbiology analyses performed at the bacteriology laboratory of the Moulay Ismail Military Hospital, Meknes, Morocco. Only isolates recovered from extraintestinal clinical specimens were included, while stool samples were excluded.

The majority of isolates originated from urinary tract infections, with additional isolates obtained from bloodstream and other extraintestinal infections. Only one isolate per patient was included in the analysis to avoid duplication.

#### 4.2.2. Broiler-Derived *E. coli* Isolates

A total of 142 non-duplicate *E. coli* isolates were recovered from broiler chickens originating from commercial poultry farms located in the Meknes region of Morocco. Necropsy and bacteriological examinations were performed at the Department of Veterinary Pathology and Public Health, Hassan II Agronomic and Veterinary Institute, Rabat, Morocco, and at a private veterinary diagnostic laboratory in Meknes, Morocco. Samples were collected aseptically from extraintestinal organs, including the liver, heart, lungs, air sacs, and yolk sac, during necropsy of broilers presenting pathological lesions compatible with colibacillosis. Sterile instruments were used, with care taken to avoid contact with intestinal contents and external surfaces to minimize the risk of environmental contamination. As apparently healthy broilers were not included, the resistance frequencies observed among isolates recovered from necropsied birds should not be considered representative of the general broiler population.

### 4.3. Bacterial Isolation and Identification

For human samples, bacterial cultures were performed on Cystine–Lactose–Electrolyte-Deficient (CLED) agar (Oxoid^®^, Basingstoke, UK) for urine specimens and on blood and chocolate agar for positive blood cultures and sterile biological fluids. Presumptive *E. coli* colonies were confirmed using Brilliance™ UTI Agar (Oxoid^®^, Basingstoke, UK) and the API 20E system (bioMérieux, Marcy-l’Étoile, France).

For avian samples, collected tissues were inoculated onto Eosin Methylene Blue (EMB) agar (Biokar Diagnostics, Allonne, France) and incubated at 37 °C for 24 h. Presumptive *E. coli* colonies were identified based on their characteristic metallic sheen and further confirmed using Brilliance™ UTI Agar (Oxoid^®^, UK) and the API 20E identification system (bioMérieux, Marcy-l’Étoile, France).

### 4.4. Antimicrobial Susceptibility Testing (AST)

Antimicrobial susceptibility testing was performed using the disk diffusion method on Mueller–Hinton agar (Biokar Diagnostics, Allonne, France). Results were interpreted according to CA-SFM/EUCAST 2024 guidelines [9].

Isolates categorized as “I” (Susceptible, Increased Exposure) were considered susceptible for statistical analysis.

To characterize presumptive β-lactam resistance phenotypes, isolates were classified according to their antimicrobial susceptibility patterns.

β-lactam resistance phenotypes were inferred from antimicrobial susceptibility patterns using predefined operational criteria based on CA-SFM/EUCAST interpretative guidance and established phenotypic characteristics of β-lactam resistance mechanisms [9,20,40].

The phenotypic classification was guided by established functional characteristics of β-lactamases, particularly their substrate profiles toward penicillins, extended-spectrum cephalosporins, cephamycins, and carbapenems [20,41].

**Figure 2:**
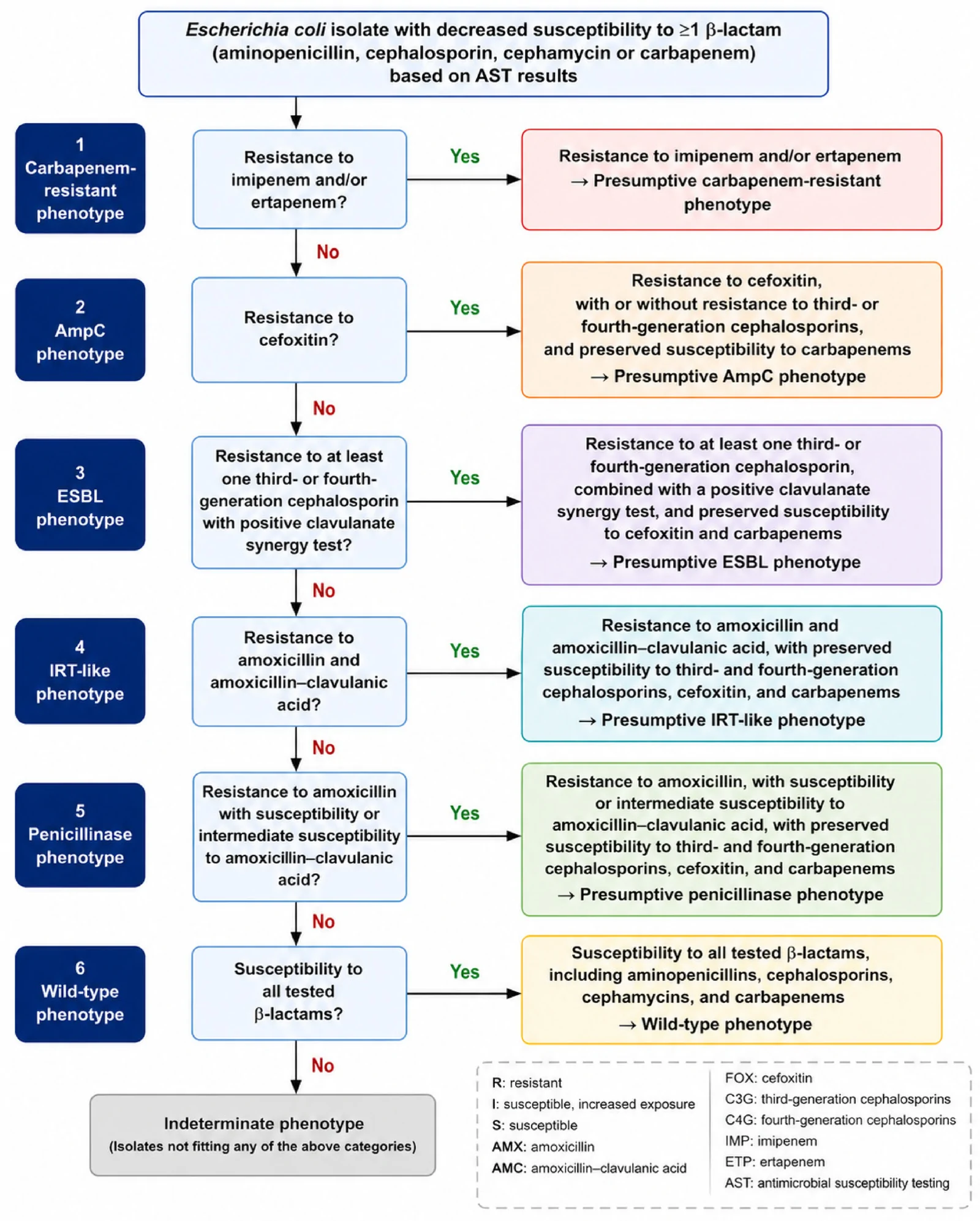
Stepwise decision algorithm for the classification of presumptive β-lactam resistance phenotypes in *Escherichia coli* based on antimicrobial susceptibility profiles. The algorithm was developed using predefined operational criteria based on CA-SFM/EUCAST interpretative guidance and established phenotypic characteristics of β-lactam resistance mechanisms. Detailed criteria are described in the Section 4.

β-lactam resistance phenotypes were assigned using a stepwise decision algorithm based on antimicrobial susceptibility patterns (Figure 2). The classification was hierarchical and prioritized phenotypic patterns associated with clinically relevant β-lactam resistance mechanisms.

The wild-type phenotype was defined as susceptibility to all tested β-lactams, including aminopenicillins, cephalosporins, cephamycins, and carbapenems [42,43].

The penicillinase phenotype was defined as resistance to amoxicillin, with susceptibility or intermediate susceptibility to amoxicillin–clavulanic acid, preserved susceptibility to third- and fourth-generation cephalosporins, preserved susceptibility to cefoxitin, and preserved susceptibility to carbapenems [20,42].

The inhibitor-resistant β-lactamase phenotype (IRT-like) was defined as resistance to amoxicillin and amoxicillin–clavulanic acid, with preserved susceptibility to third- and fourth-generation cephalosporins, cefoxitin, and carbapenems [20,42].

The presumptive ESBL phenotype was defined as resistance to at least one third- or fourth-generation cephalosporin, combined with a positive clavulanate synergy test, and susceptibility to cefoxitin and carbapenems. The clavulanate synergy test was performed by placing an amoxicillin–clavulanic acid (AMC) disc in proximity to third- and/or fourth-generation cephalosporin discs, including cefotaxime (CTX), ceftazidime (CAZ), ceftriaxone (CRO), and cefepime (FEP). A characteristic “champagne-cork” (keyhole-shaped) enhancement of the inhibition zone toward the AMC disc was considered indicative of a presumptive ESBL phenotype [9,20].

A presumptive AmpC phenotype was defined by resistance to cefoxitin, associated with resistance to at least one third-generation cephalosporin, absence of clavulanate synergy, and preserved susceptibility to carbapenems [9,20].

The carbapenem-resistant phenotype was defined as resistance to imipenem and/or ertapenem [20,44].

Isolates not fitting any of these categories were classified as indeterminate. All phenotype classifications were based on antimicrobial susceptibility patterns and phenotypic testing (including the clavulanate synergy test for ESBL detection) and should therefore be interpreted as presumptive, as no molecular confirmation was performed.

The stepwise decision algorithm used for the β-lactam resistance phenotype assignment is presented in Figure 2.

### 4.5. Antibiotics Tested

To ensure an objective comparison between human and avian isolates, several antimicrobial agents primarily used in human medicine were included in the veterinary susceptibility testing panel, including molecules not authorized for use in poultry production in Morocco [18,23].

The antimicrobial agents tested were as follows:

**β-lactams:** amoxicillin (AMX, 20 µg), ticarcillin (TIC, 75 µg), amoxicillin–clavulanic acid (AMC, 20/10 µg), cephalexin (LEX, 30 µg), cefuroxime (CXM, 30 µg), cefoxitin (FOX, 30 µg), cefotaxime (CTX, 5 µg), ceftazidime (CAZ, 10 µg), ceftriaxone (CRO, 30 µg), cefepime (FEP, 30 µg), imipenem (IPM, 10 µg), and ertapenem (ETP, 10 µg).

**Fluoroquinolones:** ciprofloxacin (CIP, 5 µg) and levofloxacin (LEV, 5 µg).

**Other antimicrobial classes:** trimethoprim–sulfamethoxazole (SXT, 1.25/23.75 µg), fosfomycin (FOS, 200 µg), and gentamicin (GEN, 10 µg).

### 4.6. Statistical Analysis

Statistical analyses were performed using IBM SPSS Statistics (version 25.0; IBM Corp., Armonk, NY, USA). Antimicrobial resistance rates were calculated separately for human and avian isolates and expressed as percentages.

Comparisons of resistance proportions between human-derived and broiler-derived isoletes were performed using Pearson’s chi-square (χ^2^) test or Fisher’s exact test, as appropriate. Odds ratios (ORs) with 95% confidence intervals (CIs) were calculated to estimate the association between isolate origin and antimicrobial resistance. An OR > 1 indicated higher odds of resistance in broiler isolates, whereas an OR < 1 indicated higher odds of resistance in human isolates.

A *p*-value < 0.05 was considered statistically significant.

Odds ratios were not calculated when zero counts were present in one group.

### 4.7. Ethical Considerations

Ethical approval was not required because the study was based on anonymized human microbiological data and bacterial isolates obtained through routine diagnostic activities. Additional antimicrobial susceptibility testing was performed on avian isolates for comparative purposes. No additional human or animal sampling was conducted specifically for research purposes.

## 5. Conclusions

This study revealed marked differences in antimicrobial resistance patterns between extraintestinal *Escherichia coli* isolates from humans and broiler chickens in Morocco. Broiler-derived isolates exhibited significantly higher resistance to aminopenicillins, amoxicillin–clavulanic acid, fluoroquinolones, trimethoprim–sulfamethoxazole, fosfomycin, and gentamicin, whereas resistance to cephalosporins was markedly higher among human-derived isolates. Resistance to cefoxitin and carbapenems was detected exclusively among human-derived isolates.

Analysis of presumptive β-lactam resistance phenotypes further demonstrated marked differences between reservoirs, with a predominance of the presumptive IRT-like phenotype among broiler-derived isolates and the exclusive detection of presumptive AmpC and carbapenem-resistant phenotypes among human-derived isolates. Overall, human-derived isolates exhibited a more diverse distribution of presumptive β-lactam resistance phenotypes, whereas broiler-derived isolates were largely dominated by the presumptive IRT-like phenotype. The detection of presumptive ESBL-producing isolates among broilers despite the absence of documented routine use of extended-spectrum cephalosporins in Moroccan poultry production may reflect the contribution of mechanisms such as horizontal gene transfer and co-selection; however, molecular investigations are required to test this hypothesis.

Further molecular studies are warranted to characterize the resistance determinants circulating in human- and broiler-derived E. coli and to assess their genetic relatedness. In parallel, studies incorporating detailed antimicrobial-use data and appropriately selected healthy-broiler populations would help clarify the selective pressures associated with the observed resistance patterns and provide a more comprehensive understanding of antimicrobial resistance across these reservoirs within a One Health framework.

## Figures and Tables

**Table 1:** Comparative frequency of antibiotic resistance in human-derived and broiler-derived *Escherichia coli* isolates.

Antibiotic	Human-Derived *E. coli* Isolates (*n*/*N*, %)	Broiler-Derived *E. coli* Isolates (n/N, %)	Odds Ratio (95% CI)	*p*-Value
**beta-lactams**				
AMX	569/869 (65.5)	132/142 (93.0)	6.96 (3.61–13.44)	<0.001
TIC	551/859 (64.1)	131/141 (92.9)	7.32 (3.79–14.14)	<0.001
AMC	410/853 (48.1)	106/141 (75.2)	3.27 (2.18–4.91)	<0.001
LEX	371/849 (43.7)	35/141 (24.8)	0.43 (0.28–0.64)	<0.001
FOX	71/870 (8.2)	0/139 (0.0)	–	<0.001
CXM	216/845 (25.6)	16/141 (11.3)	0.37 (0.22–0.64)	<0.001
CTX	169/631 (26.8)	8/137 (5.8)	0.17 (0.08–0.35)	<0.001
CAZ	165/617 (26.7)	2/140 (1.4)	0.04 (0.01–0.16)	<0.001
CRO	174/859 (20.3)	10/135 (7.4)	0.32 (0.16–0.61)	<0.001
FEP	148/581 (25.5)	1/140 (0.7)	0.02 (0.003–0.15)	<0.001
IPM	50/731 (6.8)	0/140 (0.0)	–	0.001
ETP	53/828 (6.4)	0/140 (0.0)	–	0.002
**Fluoroquinolones**				
CIP	324/853 (38.0)	108/140 (77.1)	5.51 (3.63–8.37)	<0.001
LEV	303/674 (45.0)	105/141 (74.5)	3.57 (2.38–5.37)	<0.001
**Other antibiotics**				
SXT	319/728 (43.8)	100/142 (70.4)	3.05 (2.07–4.50)	<0.001
FOS	16/379 (4.2)	33/141 (23.4)	6.93 (3.68–13.08)	<0.001
GEN	89/854 (10.4)	26/142 (18.3)	1.93 (1.19–3.11)	0.006

*n*: number of resistant isolates; *N*: number of isolates tested. Resistance rates are expressed as percentages. OR: odds ratio; CI: confidence interval. Comparisons were performed using Pearson’s chi-square test or Fisher’s exact test, as appropriate. Odds ratios were not calculated when zero counts were present. Abbreviations: AMX, amoxicillin; TIC, ticarcillin; AMC, amoxicillin–clavulanic acid; LEX, cephalexin; FOX, cefoxitin; CXM, cefuroxime; CTX, cefotaxime; CAZ, ceftazidime; CRO, ceftriaxone; FEP, cefepime; IPM, imipenem; ETP, ertapenem; CIP, ciprofloxacin; LEV, levofloxacin; SXT, trimethoprim–sulfamethoxazole; FOS, fosfomycin; GEN, gentamicin. Bold text indicates antimicrobial classes comprising multiple antimicrobial agents.

**Table 2:** Distribution of presumptive β-lactam resistance phenotypes in human-derived and broiler-derived *Escherichia coli* isolates.

β-Lactam Resistance Phenotype	Human-Derived *E. coli* Isolates n (%)	Broiler-Derived *E. coli* Isolates n (%)	Odds Ratio (95% CI)	*p*-Value
penicillinase phenotype	154 (17.6)	25 (17.6)	1.00 (0.63–1.58)	1.000
IRT-like phenotype	235 (26.9)	95 (66.9)	5.50 (3.76–8.04)	<0.001
ESBL phenotype	106 (12.1)	12 (8.5)	0.67 (0.36–1.25)	0.258
AmpC phenotype	19 (2.2)	0 (0.0)	–	0.094
Carbapenem-resistant phenotype	53 (6.1)	0 (0.0)	–	<0.001
Wild-type phenotype	289 (33.1)	8 (5.6)	0.12 (0.06–0.25)	<0.001
Indeterminate phenotype	18 (2.1)	2 (1.4)	0.68 (0.16–2.95)	1.000

Abbreviations: n, number of isolates in each phenotypic category; OR, odds ratio; CI, confidence interval; ESBL, extended-spectrum β-lactamase; IRT, inhibitor-resistant TEM; AmpC, AmpC β-lactamase. Phenotypes were inferred from antimicrobial susceptibility patterns according to the European Committee on Antimicrobial Susceptibility Testing (EUCAST) 2024 guidelines and should be considered presumptive [9]. Comparisons were performed using Pearson’s chi-square test or Fisher’s exact test, as appropriate. Odds ratios were not calculated when zero counts were present.

## Data Availability

The data presented in this study are available from the corresponding author upon reasonable request. The data are not publicly available due to privacy and institutional considerations.
